# Investigation of the Contributory Factors to the Guessability of Traffic Signs

**DOI:** 10.3390/ijerph16010162

**Published:** 2019-01-08

**Authors:** Jing Liu, Huiying Wen, Dianchen Zhu, Wesley Kumfer

**Affiliations:** 1 School of Mechanical and Electrical Engineering, Anhui Jianzhu University, Hefei 230601, China; liujing1102@ahjzu.edu.cn; 2 School of Civil Engineering and Transportation, South China University of Technology, Guangzhou 510640, China; hywen@scut.edu.cn; 3 School of Automobile and Traffic Engineering, Hefei University of Technology, Hefei 230009, China; 4 Department of Civil Environmental Engineering, The Hong Kong Polytechnic University, Hong Kong, China; 5 Highway Safety Research Center, University of North Carolina, 730 Martin Luther King Jr Blvd, Suite 300, Chapel Hill, NC 27599-3430, USA; Kumfer@hsrc.unc.edu

**Keywords:** prospective user factors, guessability, contributory factors, semantic distance, confidence in guessing, design of signs

## Abstract

Traffic signs play an important role in traffic management systems. A variety of studies have focused on drivers’ comprehension of traffic signs. However, the travel safety of prospective users, which has been rarely mentioned in previous studies, has attracted considerable attention from relevant departments in China. With the growth of international and interregional travel demand, traffic signs should be designed more universally to reduce the potential risks to drivers. To identify key factors that improve prospective users’ sign comprehension, this study investigated eight factors that may affect users’ performance regarding sign guessing. Two hundred and one Chinese students, all of whom intended to be drivers and none of whom had experience with daily driving after obtaining a license or visits to Germany, guessed the meanings and rated the sign features of 54 signs. We investigated the effects of selected user factors on their sign guessing performance. Additionally, the contributions of four cognitive design features to the guessability of traffic signs were examined. Based on an analysis of the relationships between the cognitive features and the guessability score of signs, the contributions of four sign features to the guessability of traffic signs were examined. Moreover, by exploring Chinese users’ differences in guessing performance between Chinese signs and German signs, cultural issues in sign design were identified. The results showed that vehicle ownership and attention to traffic signs exerted a significant influence on guessing performance. As expected, driver’s license training and the number of years in college were dominant factors for guessing performance. With regard to design features, semantic distance and confidence in guessing were two dominant factors for the guessability of signs. We suggest improving the design of signs by including vivid, universal symbols. Thus, we provide several suggestions for designing more user-friendly signs.

## 1. Introduction

Traffic signs, as a means of guiding travelers and transmitting road information, play an important role in traffic operation and regulation. This topic has attracted considerable interest from researchers in the past few decades. In an in-depth analysis of 77 traffic accidents, Malaterre found that neglect by road users and incorrect comprehension of traffic signs are major human errors that may result in traffic accidents [1]. Among the relevant factors, differing recognition of traffic signs among road users is a critical issue that leads to traffic accidents [2,3]. Furthermore, road users with different cultural backgrounds and languages may often be confused by traffic signs. With the growth in international and interregional travel demand, universal traffic signs may reduce the potential risks to drivers. Therefore, it is necessary to identify the universality of symbols and to consider more user-friendly designs for traffic signs for users with different cultural backgrounds.

To investigate this important issue, it is necessary to select an appropriate index to explore the usability of traffic signs. According to Jordan [4], guessability, learnability, and experienced user performance are three components that constitute the usability of a product. In addition, Jordan claimed that the guessability, measured by the cost (e.g., time and error) of using a product to perform a task for the first time, was considered a reasonable index for the readability of a product. Since users are required to recognize signs for a short time and with few errors, several previous studies [5,6,7] have used guessability to investigate the cognitive performance of users with regard to signs. Therefore, guessability was selected for further exploration in the current study.

Some studies have examined the comprehension of traffic signs by different groups [5,8]. Ng and Chan selected sign users from Hong Kong with different visiting experiences in Mainland China to explore the effects of cultural issues on the guessability of traffic signs [5]. Ou and Liu investigated the effects of design features and training on comprehension of traffic signs in Taiwanese and Vietnamese user groups and found that training experience in the relevant symbol information could significantly increase the comprehension of sign users in both groups [8]. These studies proposed that users with visiting experience in different countries/regions differ significantly in their comprehension of traffic signs. Nevertheless, few previous studies have considered users’ comprehension of signs designed in different cultural contexts [9]. As mentioned, cultural issues have been considered a significant factor that affects the usability of products [4,5]. With regard to traffic signs, there are cultural differences in design and application in different countries that may render guessability difficult in particular contexts. In the design of a traffic system, an uncontrolled junction for European drivers indicates an obligation to yield to vehicles on the right, whereas for American drivers, it represents priority for them [10]. With regard to the design of traffic signs, a previous review revealed that rectangular traffic signs provide directions or information in China, Japan, France, Spain, Germany, Singapore, Austria, Hong Kong, and Taiwan, whereas they indicate requirements, prohibitions or restrictions in America and New Zealand [11]. Therefore, it is worthwhile to investigate the effect of cultural difference on traffic sign design with regard to user performance.

Al-Madani and Al-Janahi showed that Western drivers (from developed countries) have significantly better understanding of signs compared with drivers from other countries [12]. The reason for this finding may be attributed to the difference in economic level and the development of traffic systems. Previous studies have suggested that drivers’ years of education have significant effects on their understanding of traffic signs [12,13,14]. However, few previous studies have focused on the effects of educational background for non-drivers. An-Hsiang claimed that people with different college majors show significant differences in symbol comprehension [15]. Combined with the finding of Jordan that domain knowledge is a significant factor that affects users’ performance with products [4], the guessability of signs among university students with relevant majors could be worth investigating.

A variety of studies have focused on driving experience or years of driving [12,13,16,17,18]. Al-Madani conducted several studies focusing on the effects of drivers’ personal and safety-related characteristics on their comprehension of traffic signs. These studies showed that driving experience is a significant factor [12,13,16]. More recently, Ng and Chan investigated the usability of traffic signs for inactive drivers by considering driver factors and cognitive sign features [18]. They found that the usability performance of participants who had not driven for at least a year was poor. However, none of these studies have considered users who had received driver’s license training but did not have daily driving experience, and few studies have examined prospective users who intend to become drivers. With the rapid increase in family car ownership, it has become a trend in recent years for young Chinese people aged over eighteen years to learn to drive. It is reported that there were 100 million licensed drivers in 2003, 200 million in 2010, and 300 hundred million by the end of 2014 [19], and this rapid increase in the number of young novice drivers is a notable challenge to road safety [20]. Although a previous study tested young college-aged students’ comprehension of safety signs [21], more attention to this group is needed.

Laughery et al. and Ng and Chan proposed that there was no connection between experience with traffic accidents and accurate knowledge of traffic signs [5,22]. Gender was found to be a significant factor in two studies [12,13], but other studies have found no significant effect of gender on the comprehension of signs [14,23]. Therefore, gender warrants exploration in the current study due to the contradictory findings in previous studies. The reason that the results of Al-Madani and Al-Janahi’s studies differ from other studies may be attributed to the fact that, in contrast to other studies, these authors did not control for age and level of education, which affected the comprehensibility of signs in their study.

Additionally, prior research has shown a strong relation between the degree of subjective assessment and the accuracy of cognitive tasks [24]. Thus, the issue of whether positive subjective assessment of sign guessing can affect subjects’ performance may be interesting to examine. In a previous study, subjects from vehicle-available households showed more awareness of traffic signs than did those from households without a vehicle [5]. In that study, subjects who claimed to pay attention to traffic signs in daily life performed better at sign guessing than those who did not [5]. Therefore, we aimed to explore the effects of these two factors in the current study.

With regard to the impact of sign features, cognitive features such as familiarity, concreteness, simplicity, meaningfulness, and semantic distance are of central concern in sign research. Some previous studies have used these five sign features to investigate the usability or guessability of traffic signs [7,8,14,17,23,25,26]. More specifically, Liu conducted a two-stage simulation experiment to investigate the effect of drivers’ viewing strategies and sign familiarity on performance in visual search [17]. Subjective workload evaluation indicates that drivers with less sign familiarity will be under greater time and visual pressure. Ng and Chan designed several studies focusing on the effect of these five design features on users’ comprehension performance. Their results showed that semantic closeness was the best predictor among these features [23,25]. Chan and Chan investigated the effects of prospective user factors and sign design features on the guessability of pharmaceutical pictograms and also used these five features [7].

Familiarity is the frequency with which signs have been encountered in the past. A concrete sign has an obvious connection with the real world. Signs are regarded as complex if they contain many details or are intricate. Meaningfulness refers to how meaningful a sign is perceived to be. Semantic distance is the distance of the relationship between what is depicted on a sign and what it is intended to represent. Sharples et al. highlighted the role of familiarity with information wording and context in drivers’ trust in the information of variable message signs. This is an important factor in evaluating the guessing performance of users [27]. McDougall found that icon complexity did not have a close correlation with the other features, but it played an important role in the guessability of signs [28]. Many relevant studies have claimed that semantic distance is the best predictor of sign comprehension [6,8,23,26]. In combination with the results of these prior studies, the current study considered complexity and semantic distance. With regard to the other two features, previous research has demonstrated interrelationships among concreteness, meaningfulness, and semantic closeness, with the meaningfulness of a stimulus depending on its familiarity and associated imagery [25]. This finding was consistent with McDougall’s study [28]. Thus, meaningfulness and concreteness were not considered suitable for this investigation because they share considerable variance with semantic closeness, which means that the function they play can be replaced by the function of semantic closeness. Therefore, familiarity, complexity and semantic closeness are better options.

The current study has four objectives: (1) Cultural issues were considered to identify the differences between the performance of Chinese users in guessing Chinese signs and German signs. (2) User factors were investigated to determine the relevance of gender, vehicle ownership, grade, driver’s license training, living area, attention to traffic signs, traffic incident experience, and subjective self-assessment to sign guessability. (3) Cognitive features, including familiarity, complexity, and semantic distance, were examined to verify their effects on the guessability of signs. Additionally, given that previous studies have suggested that comprehension is an interactive and dynamic process and that subjects’ confidence may affect their sub-conscious [29], a new feature reflecting users’ cognitive process was developed, “confidence in guessing”. We included this feature to evaluate whether users’ confidence affects their understanding of the meaning of signs. (4) The findings were synthesized to identify contributing factors to provide guidance for improving sign guessability.

## 2. Method

A rating scale was designed for the experiment, and subjects were selected to participate in a questionnaire survey. Information on user factors and the sign-guessing performance of the subjects was collected in the questionnaire. The participants were required to guess the meaning of signs and to provide rating scores for these features.

### 2.1. Participants

A total of 207 students who were transportation engineering majors from one university in mainland China volunteered to participate in the survey. The participants included 109 males and 98 females ranging in age from 19 to 23 years old (age: MEAN = 20.32, SD = 1.232). We recruited the participants by communicating with their class monitors. The participants were informed that the experiment was anonymous and that it was conducted for academic research and was not related to do with their own interests. Each participant in the survey was compensated with a reward of 10 yuan. All of the participants were reported to have normal or corrected-to-normal vision. None of them had received traffic sign training during their college education or had visited Germany, and none of them had daily driving experience, although some of the participants had Chinese driver’s license training.

### 2.2. Instruments

A color-blindness test paper was prepared to test the candidates’ vision. We used a 15-inch personal computer, an Epson projector and a projection screen to conduct this experiment. Additionally, a conference room that could accommodate 25 students was prepared.

### 2.3. Traffic Signs

To conduct the survey, we selected appropriate traffic signs as samples. The considerations for selection were as follows: 1. the traffic signs must convey information only by images and numbers (therefore, most of the guide signs were removed); 2. signs whose key information needed to be conveyed by other signs were excluded; and 3. the number of Chinese signs was larger than the number of German signs because our main analysis focused on the guessing performance of prospective users for Chinese signs, although some German signs were used as a comparison group for the investigation of cultural issues.

Finally, 39 Chinese mainland traffic signs from *The Road Traffic Signs and Markings* (GB 5768-2009, issued in April 2009) and 15 German signs (selected from BMVI, the official website of the German transportation department) were selected for this investigation. The specific signs are shown in Table 1. The signs numbered 14 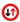, 15 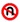, 25 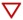, 29 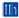, 30 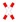, 31 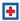, 32 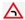, 35 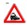, 37 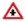, 38 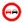, 40 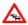, 43 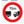, 52 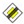, 53 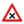, and 54 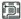 were German signs. Some traffic signs in China are very similar to the same signs in German due to the Vienna Convention on Road Traffic. Thus, we told the participants not to consider factors unrelated to the symbol.

### 2.4. Questionnaire and Sign Feature Evaluation Sheet

#### 2.4.1. Prospective User Factors

A questionnaire with eight closed-ended questions was designed to capture personal information (i.e., prospective user factors). The first question was about the subjects’ college grades. The subjects checked boxes for year one, two or three. Students who selected year one were regarded as freshman college students (marked as grade 1), students who selected year two were regarded as sophomores (marked as grade 2), and students who selected year three were regarded as junior students (marked as grade 3). The next six questions were as follows: (1) Do you have driver’s license training experience? (2) Do you or your family own a vehicle? (3) Have you or a family member been involved in a traffic accident or incident in the past two years? (4) Do you live in an urban area? (5) Do you usually pay attention to the design of traffic signs in daily life? (6) Do you think you can guess the information by yourself? The final question was about gender and required the subjects to check the “male” or “female” box.

#### 2.4.2. The Rating Scores of Signs along Different Scales

This part of the questionnaire included columns for sign patterns, familiarity, complexity, confidence in guessing, semantic distance and sign guessing. Based on a Likert scale [30], the ratings were classified into five levels (shown as Table 2). For familiarity, the number 1 indicated completely unfamiliar and the number 5 corresponded to very familiar. For complexity, the number 1 indicated completely simple and the number 5 corresponded to very complex. For confidence in guessing, the number 1 indicated completely unconfident and the number 5 corresponded to very confident. For semantic distance, the number 1 indicated completely consistent and the number 5 corresponded to completely inconsistent. In the sign-guessing column, the subjects were required to write their guess of the sign’s meaning. All the 5-point scores were transformed into a percentage system to map the interrelationships among the design features as well as the relationship between the feature rating score and the guessing score.

### 2.5. Procedure

The subjects were given three minutes to complete the section on personal information and to review the entire questionnaire. In the first stage, they were given sufficient time to complete the rating columns for each sign, with the exception of the “semantic distance” column. In the second stage, the correct meanings of all the signs were given to the subjects. They rated “semantic distance” by comparing the true meaning with the symbol’s cognitive meaning in the next stage without changing their written answers. After removing questionnaires with incomplete responses, a total of 201 questionnaires were collected.

### 2.6. Analysis

**Figure 1:**
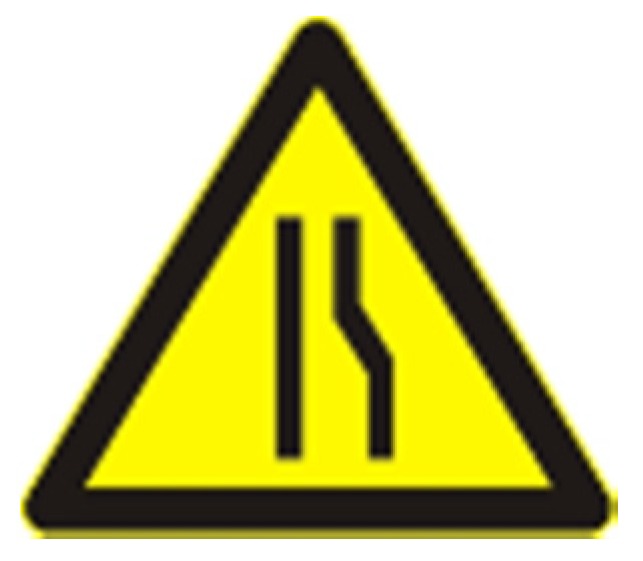
A sample to show the evaluation process.

The 0–3 level system was chosen to examine the feedback of the subjects. An answer that was fully consistent with the given meaning of the sign was given 3 points (absolutely correct). If the answer was not exactly the same as the original meaning but was similar to it, it was given 2 points (nearly correct). An answer that deviated from the given meaning but was partially consistent with it was given 1 point (partially correct). An answer that was completely unrelated to the given meaning was marked 0 (completely wrong). The correctness of answers was judged on the basis of whether the subject’s answers included key information or expressed the main meaning (e.g., for the sign shown in Figure 1, the correct meaning is “pay attention to the road ahead, which narrows on the right”; if a subject answered “the right side narrows”, that answer received three points; “the road ahead narrows” received two points; and other answers received 0 points). Measures were taken to avoid subjectivity in the assessment. Answers were evaluated by an objective assessment team composed of three postgraduate students and a teacher to determine the key response components required for each point valuation per question. The authors then compared their assessments, and controversial results were resolved by discussion. In the data analysis section, the 3-point guessing score is transformed into a percentage system to be consistent with the dimensions of the sign feature rating score.

With regard to the data processing, descriptive statistics and a one-sample Kolmogorov–Smirnov test were used to find the general distribution of the data. To investigate the effects of each user factor on users’ guessing performance, we used an ANOVA (when the data of a factor were normally distributed) and the Kruskal–Wallis test (when the data of a factor were not normally distributed) to investigate the differences in guessing performance between the subject groups within each factor. Since there may be interrelationships among the eight user factors, an interaction effects test was conducted to determine these interrelationships. For each statistically significant interaction effect, main effect contrasts were developed to examine the effect of one factor at each level of the other factors. For the signs’ cognitive features, correlation analysis and regression analysis were conducted to investigate the interrelationship among four features and the relationship between these features and the guessability of signs. After determining the degree of correlation between these features and the guessing scores, a partial correlation analysis was conducted to find the role each feature played in the guessing score of the signs.

In addition to the statistical processing in the factor analysis, we attempted to find useful information for sign design. First, signs whose guessed scores had high levels of variability were selected. Then, according to the subjects’ responses to these signs, the reasons for these extreme scores were analyzed. Furthermore, we investigated the performance of Chinese subjects with Chinese and German signs and explored the effects of learning experience with Chinese signs. Finally, combining the cognitive features that significantly influenced the guessability of the signs, we compared the design of these two categories of signs and provided suggestions to improve the guessability of signs.

## 3. Results

The overall guessing score of one subject reflects the subject’s guessing performance in this task, and the average guessing score of one sign reflects its guessability level.

### 3.1. Descriptive Statistics for Guessing Scores

**Figure 2:**
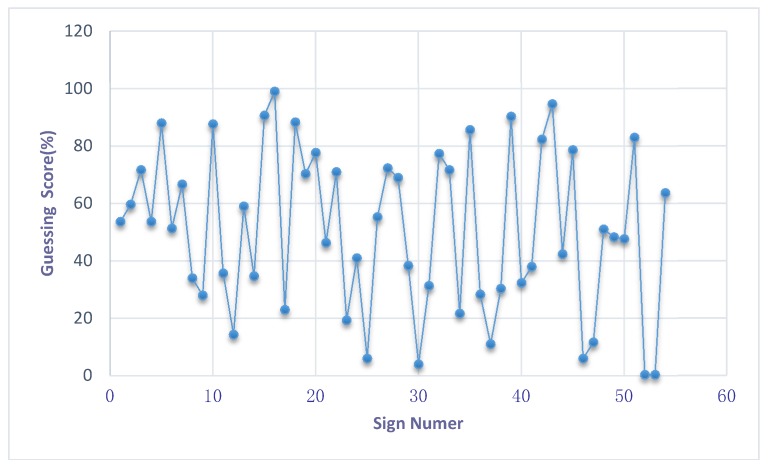
Guessing score distribution of all selected sample.

The general guessing score distribution of all selected samples is presented in Figure 2. A one-sample Kolmogorov–Smirnov test showed that the guessability scores were approximately normally distributed (Table 3). The fit of the normal state was significant, and the mean score was 50.67% (1.52 points in a 3-point system; we transformed the 3-point system into a percentage system accordingly). The distribution ranged from nearly 0% to 99%, whereas 74% (40/54) of the sign-guessing scores were distributed from 20% to 80%. The 54 signs selected for this experiment could be classified into six major categories according to Road Traffic Signs and Markings (GB 5768-2009) as shown in Table 4. Warning, prohibiting and mandatory signs constituted the main part of the sample because these three types of signs account for a large proportion of the current traffic sign system. Most guide and tourist signs were excluded due to consideration 1 mentioned in Section 2.3, and most roadwork signs were excluded due to additional environmental clues, such as visible construction and roadwork barrels, which help drivers to interpret these signs. Some signs that could not be classified into these six major categories were classified into a special category (numbered 30 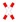, 31 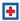, 37 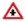, 52 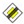 and 53 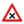). Warning signs had the highest average guessing score (62.88%), whereas the average score for special category signs was the lowest (9.4%). The average guessing score for prohibition signs was 57.09%, but their standard deviation was the highest, indicating large differences among those samples.

### 3.2. Signs with Extremely Variable Scores

**Figure 3:**
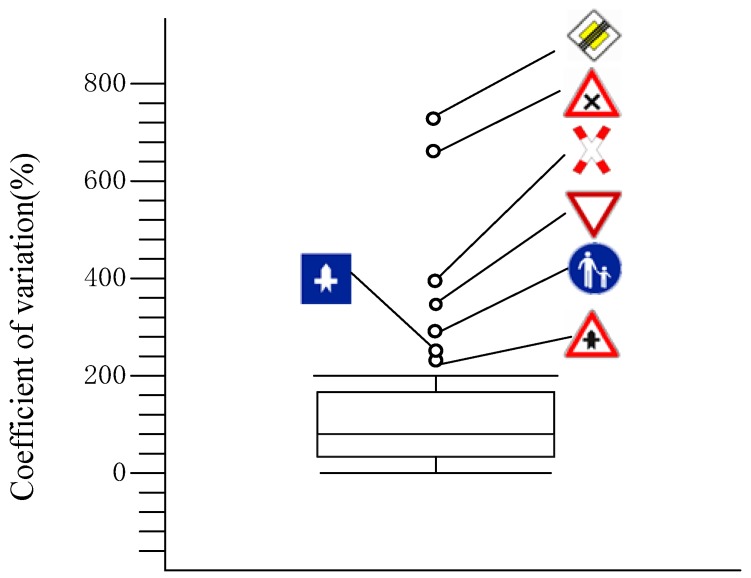
Box plot of coefficients of variation on guessability score.

To determine whether there were any signs whose variability in the guessability score was considerably different from other signs, a box plot of the coefficients of variation in the guessability score for all signs was prepared (Figure 3). Coefficients of variation were chosen as the evaluation indicator for the variability of the guessability score because it is a dimensionless quantity and does not require data on the mean value. The criterion for evaluation was that values more than 1.5 box lengths (difference of 75th and 25th percentiles) away from the box would be regarded as outliers [31].

The signs numbered 25 (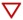), 30 (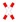), 37 (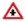), 46 (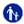), 47 (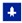), 52 (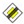), and 53 (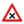) were assessed as outliers above the box (specific information on these signs is shown in Table 5), indicating that their measure of dispersion was much higher (over 50%) than the other signs, whose coefficients of variation ranged from 8.70% to 145.81%. The guessed scores of the nine signs are also shown in Table 3, including the three most frequent responses to each sign. The signs whose measure of dispersion was much higher than others (25 (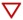), 30 (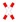), 37 (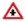), 46 (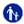), 47 (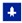), 52 (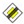), and 53 (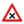)) also received the lowest guessability scores.

According to the three most frequent responses for signs rated with low scores (shown in Table 5), signs 25 (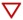) and 52 (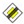), which were German signs, received more than 50% “do not know” responses, which indicates that the subjects did not know how to begin the guessing process. The most frequent response for signs 37 (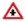) and 47 (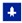) was “rocket.” This finding indicates that the designed symbol was regarded as a rocket, which did not match the true meaning (“Traffic has priority in the main road”). It is surprising that sign 46 (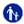) received such a low rating score because it is a common sign in school and residential areas. Furthermore, this sign received a high familiarity score (48.82%), a high confidence in guessing score (46.85%) and a low complexity score (42.42%). Most responses to this sign were concerned with “children” or “school.” It can be inferred that the symbol was regarded as a man holding his child’s hand rather than a place for pedestrians only, and this may explain why the rating score for semantic distance was large (68.41%).

### 3.3. The Relation between the Guessing Performance of the Subjects and Prospective User Factors

#### 3.3.1. Analysis of Variance and K-W Test for the Prospective User Factors

Table 6 shows the prospective user factors, the number and percentage of the subjects’ group divided by their response to each factor, and the guessing performance of the subjects in the response categories.

To test the hypotheses that user factors affect sign guessability performance, an analysis of variance (ANOVA) and a Kruskal–Wallis test for within-subjects factors were conducted. The results are shown in Table 7. The guessing performance for subjects was in the range of 31.10–85.69%, with a standard deviation of 10.92%. Subjects’ guessing performance had a very high inter-rater reliability coefficient (0.94), indicating a high level of consistency among the judges.

Factors including driver’s license training and traffic incident experience were normally distributed (Shapiro-Wilk test, *p* > 0.05), whereas the other six factors were not. In addition, the variances at all the levels were equal (Levene’s test, *p* > 0.05) for all eight factors. Therefore, the effects of the first two factors on guessing performance were examined with an ANOVA (significant threshold, *p* < 0.05), and the rest were analyzed with a Kruskal–Wallis test (significant threshold, *p* < 0.005).

The presence or absence of driver’s license training had a significant impact on sign guessing (*F* = 266.66, *p* < 0.05). Subjects with driving skill learning experience showed significantly higher average guessing scores (61.06%) than did those without a driver’s license (43.98%), which could be attributed to the fact that driver’s license training includes knowledge of traffic signs. Whether the subject’s family had a vehicle had a significant impact on guessing performance (χ^2^ = 8.08, *p* < 0.01). The average guessing score (53.04%) of families with a car was higher than the average score of those without a car (49.27%). The experience of travelling with a car or having a vehicle is likely to improve travelers’ awareness of sign information, leading to better guessing performance. Gender did not have a significant effect on guessing performance (χ^2^ = 1.59, *p* = 0.201). Paying attention to the design of traffic signs also had a significant impact on sign guessing (χ^2^ = 16.751, *p* < 0.005). The average guessing score (54.35%) of subjects who showed a deliberate focus on traffic signs was significantly higher than the score of those who did not (47.23%). Experience with traffic accidents did not show a significant impact on sign-guessing performance (*F* = 0.16, *p* = 0.69). Guessing performance among the three levels of grades showed significant differences (χ^2^ = 44.435, *df* = 2, *p* < 0.005), and the overall guessing performance increased with an increase in grade level (grade 1 < grade 2 < grade 3). Subjects who lived in urban areas had better (but not significantly better) guessing performance than those living in rural areas (χ^2^ = 3.276, *p* = 0.095). Whether subjects believed that the sign meaning could be guessed only by themselves did not show a significant effect on guessing performance (χ^2^ = 2.33, *p* = 0.55).

#### 3.3.2. Analysis of Interaction Effects among User Factors

Since there may be interactions and correlations among user factors, we conducted a two-way interaction effects test to explore this issue. There were 28 interactions between each of the factors. The significant interactions were having driver’s license training experience and vehicle ownership (*F* = 6.42, *p* < 0.05); having driver’s license training experience and subjective assessment (*F* = 4.93, *p* < 0.05); grade in college and subjective assessment (*F* = 4.69, *p* < 0.05); and attention to the design of traffic signs and subjective assessment (*F* = 7.72, *p* < 0.05).

For each statistically significant interaction effect, main effect contrasts with ANOVA between-subjects factors were conducted to examine the effect of one factor at each level of the other factors [32,33]. The results showed significant differences in guessing performance between subjects from vehicle-unavailable households with driver’s license training experience (62.02%) and those without driver’s license training (42.84 %) [*F* = 194.38, *p* < 0.005]. There was also a significant difference between the two driving learning experience groups from vehicle-available households (having = 58.74%, not having = 44.97%) [*F* = 71.62, *p* < 0.005]. Among subjects who did not believe that the meaning of signs could be guessed only by patterns, the group with driver’s license training (57.62%) showed a significant difference from the other group (43.59%) [*F* = 78.04, *p* < 0.005]. The two different experience groups of subjects who had confidence in guessing performance (having = 61.97%, not having = 43.32%) also showed a significant difference (*F* = 193.41, *p* < 0.005). Grade in college was found to be a significant factor for subjects who provided positive subjective assessments (grade 1 = 42.72%, grade 2 = 54.32%, grade 3 = 55.84%, *F* = 19.92, *p* < 0.005). There was a significant difference between subjects who provided a positive subjective assessment with attention to traffic signs (56.14%) and those without attention to traffic signs (45.861%) [*F* = 28.75, *p* < 0.005]. There was no significant difference between subjects with negative subjective assessments with attention to signs and those without attention to signs (*F* = 0.99, *p* = 0.46).

### 3.4. Signs’ Cognitive Features

#### 3.4.1. Interrelationships among Traffic Sign Features

**Figure 4:**
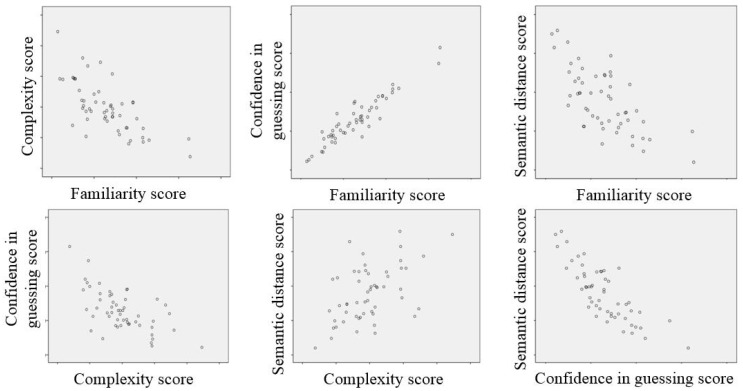
Scatter plots for the relationships between the pairs of features.

A correlation analysis was used to test the interrelationships among the sign features. Green and Salkind specified three assumptions underlying the most widely used indicator of correlation [33], the Pearson correlation coefficient: (i) two sets of data are normally distributed; (ii) the relationship between the two sets of data is linear; and (iii) each pair of data is independent from all other pairs. The ratings on the four sign features were normally distributed (Shapiro-Wilk test, *p* > 0.05), and the general relationship between each feature is shown in Figure 4. Table 8 shows the result of the correlation analysis, in which confidence in guessing was highly correlated with familiarity (*r* = 0.935, *n* = 54, *p* < 0.005) and semantic distance (*r* = −0.813, *n* = 54, *p* < 0.005). Familiarity was strongly correlated with complexity (*r* = −0.701, *n* = 54, *p* < 0.005), which showed the weakest correlation with semantic distance (*r* = 0.519, *n* = 54, *p* < 0.005). Familiarity showed a general correlation with semantic distance (*r* = −0.689, *n* = 54, *p* < 0.005) and with complexity and confidence in guessing (*r* = −0.622, *n* = 54, *p* < 0.005).

#### 3.4.2. Relationships among Traffic Sign Features and Guessability Score

**Figure 5:**
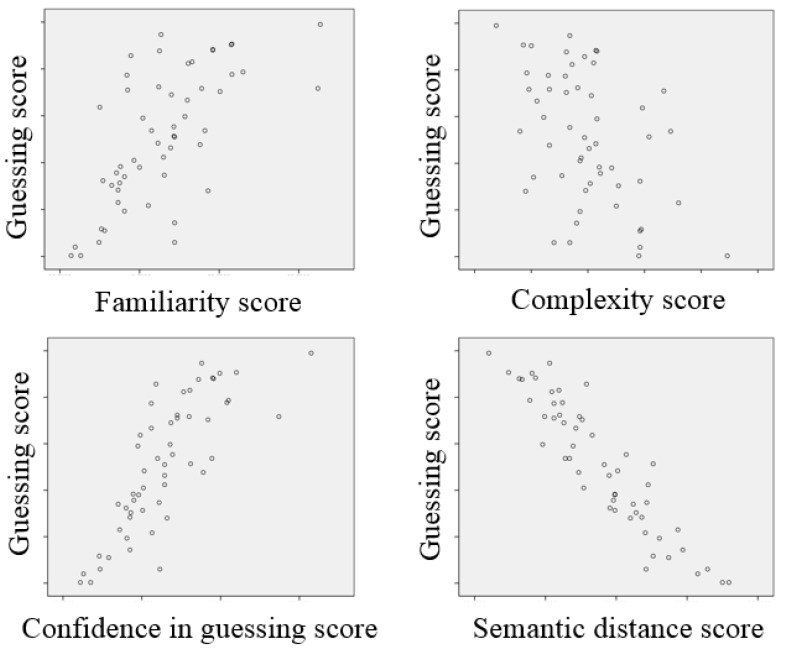
Scatter plots for the relationships between the cognitive features and sign guessing score.

The relationships between sign-guessing scores and sign features were approximately linear according to the scatter plots (shown in Figure 5). After eliminating the few extreme points, Pearson correlation analysis was conducted, and signs whose coefficients of variation on each feature were much higher (50% higher) than others that were excluded. The results indicated that the four sign features and the guessability of traffic signs were significantly correlated (as shown in Table 9). Semantic distance showed the strongest correlation with the sign guessability score (*r* = −0.923, *n* = 53, *p* < 0.005), whereas the relationship between complexity and the guessing score was weakest (*r* = −0.423, *n* = 50, *p* < 0.005). Confidence in guessing showed a high correlation with the guessing score (*r* = 0.820, *n* = 51, *p* < 0.005), with familiarity correlating with the guessing score at a general level of significance (*r* = 0.672, *n* = 52, *p* < 0.005).

By taking the four traffic sign features as the independent variables, a multiple regression model analysis of the 54 signs was conducted. The results are as follows:

(1) $$\begin{array}{l} predicted\;guessing\;\mathrm{score}(\%)=2.847-0.327\;\mathrm{familiarity}+0.066\mathrm{complexity}\\ +0.646\mathrm{confidence}\;\mathrm{in}\;\mathrm{guessing}-0.796{\left(\mathrm{semantic}\;\mathrm{distance}\right)}^{2} \end{array}$$

The prediction model showed a significant fit to the data (*R*^2^ = 0.933, *p* < 0.05). Nevertheless, unexpected abnormal coefficients for familiarity and complexity were found, and the multiple regression weights were not interpretable, indicating the possible existence of a problem with multi-collinearity among the independent variables [34].

To verify this problem, a collinearity test was conducted with four indicators: the simple correlation coefficient, variance inflation factor, eigenvalue, and condition index. The results showed that collinearity was slightly serious because two variance inflation factors of each sign were more than 10 and one of the condition indexes was more than 30. Collinearities generally occurred due to internal relations among the variables. Thus, when the sign features were regarded as variables, familiarity, confidence in guessing and semantic distance were not completely independent.

To resolve the collinearity that appeared among the features, partial correlation analysis was conducted to identify the most useful feature. By excluding the other three factors, the results of the partial analysis showed that semantic distance was the best feature for the guessability score (*r* = −0.744, *p* < 0.005), followed by confidence in guessing (*r* = 0.307, *p* < 0.05). Familiarity and complexity did not show significant impacts (*r* = −0.207, *p* = 0.146; *r* = 0.061, *p* = 0.673).

### 3.5. Analysis of Subjects’ Guessing Performance on Signs from Two Countries

To verify whether the learning experience for Chinese signs affected guessing performance on signs from another country (Germany), further analysis was conducted to explore the guessing performance of subjects on German and Chinese signs. Although the 15 German signs were completely unfamiliar to the subjects, the average score for guessing the 15 German signs of subjects with a driver’s license was 39.98%, which was significantly higher than the score of those without a driver’s license (29.48%) (*F* = 81.90, *p* < 0.005). This finding may indicate that the experience of obtaining a driver’s license or learning traffic signs in China could potentially improve understanding of German signs. For the 41 Chinese signs, the overall guessing performance of subjects who had driver’s license training experience (79.00%) was significantly higher than the performance of those without this experience (41.29%) [*F* = 322.42, *p* < 0.005], which indicates the significant effect of learning experience. Furthermore, the results showed that the overall guessing score of Chinese signs was significantly (68.06%) higher than that of German signs (34.28%) [*F* = 274.25, *p* < 0.005]. However, since a direct comparison between sign meanings was not possible, further research is needed to evaluate whether these findings are generalizable (i.e., whether receiving driver’s license training and driving experience in one region improves guessability in all other regions).

## 4. Discussion

### 4.1. Prospective User Factors

The gender and traffic incident experience of the subjects and their families showed no effect on their guessing performance. The finding about gender was consistent with most previous studies [14,23], except the studies by Al-Madani and Al-Janahi. As mentioned previously in the Introduction, the reason for the inconsistency between Al-Madani and Al-Janahi’s studies and other studies may be that we also controlled for age and level of education, which are considered significant factors, and cultural difference may be an important reason for that inconsistency. The result for the effects of traffic incident experience was consistent with previous studies [16,22,23]. This may indicate that traffic accident experience did not improve awareness of traffic signs for those subjects. Thus, it may be necessary for the Department of Transportation to strengthen inductive education on traffic signs, similar to traffic security education.

Contrary to expectations, the designed factor of subjective assessment showed no significant effects on subjects’ guessing performance. In terms of living area, we assumed that people living in urban areas should perform better due to better transport facilities and more opportunities to encounter signs [35]. However, this factor showed no significant effects on the subjects’ guessing performance.

Whether the subject’s family owned a car had a significant effect on the subject’s guessing performance. Subjects whose family owned a car had better overall guessing performance than those whose families did not, suggesting that the experience of traveling in a car can improve travelers’ cognition of road traffic signs. The finding is contrary to the findings of a prior study [23]. However, our finding may be more acceptable for the following reasons: travelers can obtain access to traffic signs in a real-world environment, which may improve their understanding of the relationship between traffic signs and real circumstances [14], and traveling in private cars may make people pay more attention to road signs since attention to traffic signs also showed a significantly positive influence on guessing performance. However, in Ng and Chan’s study [23], the experience of travelling in a car showed negative effects on travelers’ cognition of road traffic signs, which was also contrary to their original expectation. They hypothesized that the effects should be positive, and the reasons they claimed for the hypothesis were similar to our analysis. Furthermore, a recent study proposed that travelling in a car could help to improve users’ attention to traffic signs [36], which potentially supports our findings due to the significant positive effects of attention to signs shown in the current study and in Ng and Chan’s study.

According to the interaction effect test, subjects who paid attention to traffic signs in their daily life performed better at guessing signs than those who did not. This finding is consistent with previous studies [13,23]. It seems that people who pay attention to the design of signs in daily life have a tendency to perceive and recognize the functions of traffic signs. For the group that provided a negative assessment with guessing, attention to signs showed no significant effect on guessing performance.

### 4.2. Signs’ Cognitive Design Features

With regard to sign familiarity, signs with higher familiarity were guessed more easily, but this may not be the most important factor in judging the guessability of a sign. What is familiar to one person may not be familiar to another. Shinar et al. and Alan Chan found that infrequently encountered signs are more likely to be miscomprehended and are less likely to be correctly learned by drivers [23,37]. Rosson noted that designers should use familiar symbols as much as possible [38]. The role of familiarity was verified because the results of the correlation analysis showed a more significant relationship with the sign-guessing score than with complexity. However, when the interaction between factors was excluded in the partial correlation analysis, the result showed no significance between familiarity and the guessing score. In addition, we found that confidence in guessing and familiarity shared extremely high variance (92.5%) through the correlation analysis, which may indicate that the role of familiarity can be replaced by confidence in guessing, although determining a comprehensive rating for confidence in guessing may be more difficult.

Confidence in guessing is a designed feature based on the theory of cognitive psychology [39]. The results of the correlation analysis showed a high correlation (82.3%) between confidence in guessing and the score for sign guessing. The results of the partial correlation analysis for confidence in guessing also showed a significantly positive effect on sign guessing, which suggests that confidence in guessing can be considered an important factor to assess the guessability of signs

With regard to complexity, previous studies have found that simple symbols are more easily understood than complicated ones [23,40,41]. However, we found that complexity contributed the least of all four features, and the relationship between complexity and the guessing score was not significant when the effects of the other three features were excluded. Hence, it is not always the case that lower complexity leads to better guessability. For instance, signs 25 (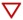) and 53 (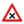) were regarded as simple designs, but they received the lowest guessing scores, with most subjects responding “Do not know.”

Previous studies have proposed that semantic distance (contrary to semantic closeness) plays the largest role in the guessability of a sign. In this paper, the Pearson correlation analysis and partial coefficients analysis both confirmed that semantic distance was the best predictor of the guessability of signs. Given the confirmation of the importance of semantic distance, this may be regarded as the most important design principle and thus may provide a criterion for the evaluation of the guessability of traffic signs.

### 4.3. Analysis of Contributory Factors

In a previous study, Ng et al. confirmed that the contributions of the five sign features to guessability were not equal, and semantic closeness was by far the best predictor of guessability [23]. In addition, McDougall et al. found that semantic proximity was more important than validity, specificity and easiness in understanding a fixed sign [42]. However, previous studies did not consider features related to the cognitive process. In addition, few previous studies have identified contributory factors or provided specific advice on sign design based on the research findings. Therefore, we attempted to conduct an analysis of contributory factors and provide some suggestions on design improvements (shown in Section 4.4).

As expected, driver’s license training showed positively significant effects on guessing performance at each level for both groups with and without vehicle ownership. Thus, it can be inferred that compared to vehicle ownership and subjective assessment, driver’s license training showed a more significant contribution to guessing performance. Furthermore, grade in college also showed significant effects on guessing performance.

Therefore, we identify the first contributory factor as training experience. Both learning experience and grade in college (major in transportation) indicate the dominant effects of experience and training in relation to traffic knowledge, such as traffic rules, urban planning and speed control methods. Furthermore, the signs that are currently used (at least those we chose) were difficult for novices and inexperienced road users to comprehend. Therefore, the design of the selected signs should be improved.

With regard to signs’ cognitive design features, complexity was confirmed as an unimportant factor for sign design, and the contribution of familiarity can be replaced by confidence in guessing. Semantic distance and confidence in guessing are two contributory factors for the guessability of traffic signs. To reduce semantic distance, we recommend that the elements of symbol design should match the conveyed messages. Confidence in guessing can be used as an effective index for evaluating the reasonability of signs.

### 4.4. Cultural Issues and Suggestions on Design Improvement

**Figure 6:**
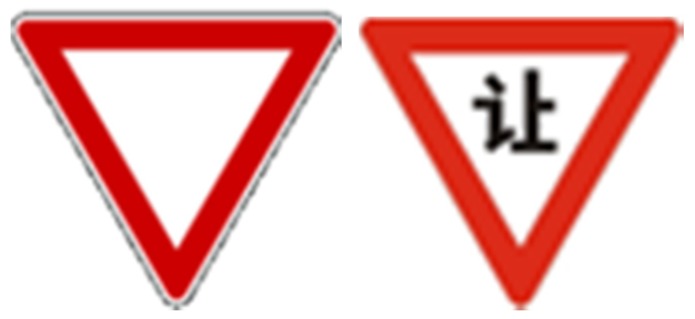
Two signs convey the same meaning: “slow down and yield to others.”.

Since the overall guessed score of Chinese signs was much higher than that of German signs, we conducted a further analysis to identify potential reasons for this result. With the exception of signs from both countries with similar symbols (such as 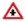 (German) and 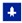 (Chinese)), considering the positive effects of learning experience of Chinese signs on subjects’ guessing performance for German signs, a comparison was made between the German signs with low- and high-guessed scores (none of the subjects had experience visiting Germany or experience learning German signs). Signs 25 (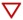), 30 (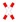), 37 (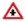), 52 (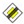), and 53 (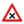) received the lowest level of guessability scores (less than 10%), whereas signs 43 (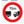) and 54 (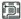) received extremely high-level scores (94.67%, 63.67%). The common characteristics of signs with high-guessed scores were a low semantic distance rating score and high visualization of conveyed information. For instance, sign 43 showed a “3 m” between two trucks, and the edge of the sign was red, which indicated a warning for the distance between trucks. Therefore, the meaning of this sign could be easily understood as “Watch out! the distance should not be less than 3 m.” Signs 52 and 53 received extremely low scores (0.33%). The images used on these signs did not match the meaning they actually represented. Specifically, the meaning of sign 52, “the end of priority road,” is related specifically to the German cultural environment, and it is nearly impossible for foreigners without visiting experience to understand the meaning as “main road” or “end.” Thus, a cultural issue was found in this aspect, which indicates a specific image that can be recognized only by people with a particular cultural background. A previous study recommended that text explanations could also be used when cultural bias is present or the meanings of signs are difficult to convey [25]. Nevertheless, symbols rather than text are a common way of conveying information to different groups of users. We recommend the use of symbols only when a cultural issue needs to be expressed. For example, two signs (sign 25 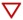 and sign 33 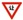) conveying the same meaning are shown in Figure 6. A substantial distance between the rating scores for these two signs was found: sign 33 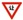 received a guess score of 71.67%, whereas sign 25 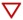 received a guess score of only 6%. The designs of the two signs were identical (both were designed as an inverted triangle), which means that the symbol was not the reason for the difference. It is obvious that the Chinese text in sign 33 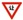 contributed substantially to users’ comprehension of the sign, suggesting that an explanation of the symbol helps with the cognitive process. Furthermore, traffic signs in Japan widely use local texts to convey information. However, foreigners who could not understand the text could not guess the meaning of sign 33 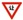 by recognizing the meaning of the symbol, just as Chinese subjects could not understand the meaning of sign 25 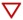 because the inverted triangle does not match information on yielding. Adding the meaning of the sign in writing also has a disadvantage because it adds to the sign complexity.

To alleviate the cultural issue, we provide several suggestions to improve the design of signs with low guessing scores (shown in Table 10). Generally, we recommend using more vivid symbols during the design period. More specifically, we recommend the use of icons or shapes that are closely connected with the real environment. A previous study suggested that concrete information, such as distances or lane configurations, can reduce the probability of making errors when people are performing cognitive work [43]. Taking the first sign (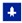) as an example, we note the key point that the thicker the lines, the higher the road rights. We attempted to reduce the misunderstanding of “rocket,” so we designed a new sign (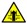) in which we drew the road and the rights line separately.

Another experiment to evaluate the redesign of signs was conducted, and rating scores were identified for improved signs rated by 24 students. The results showed that confidence in guessing was higher after redesign, and a *t*-test showed that the guessing scores were significantly improved (*p* < 0.05).

Signs generally convey their own meaning, and text usually adds information rather than clarifying the meaning of the sign. A prior study claimed that driving speed could also influence users’ cognition of signs under real environmental conditions [44]. This study focused on the meaning conveyed by symbols and investigated users’ cognitive work in a simulation environment.

## 5. Limitations

Limitations to the experimental design were also identified. First, the participants’ personal information was completed on the spot. Due to social desirability, the information the participants provided may not have been completely accurate, especially for items related to privacy, such as home address and whether the participant had a car. Second, compared with previous studies, this experiment included more participants, but we reduced the number of traffic signs for the following reasons. We chose representative signs based on previous studies and a pre-experiment; therefore, the guessability scores of the chosen signs were normally distributed. Furthermore, we used 200 signs to conduct a pre-experiment, but the time spent on the guessing and rating tasks was too long and made the participants impatient to complete the experiment. Finally, we did not apply the “improvements” in a real environment, although they seem beneficial based on the lab experiment. It should also be noted that the sample sizes of the signs from China and Germany were different. Therefore, a direct comparison between the guessability of the two sign types was not suitable in the current study.

## 6. Conclusions

This experiment demonstrated the effective relationships among user factors, signs’ cognitive features and guessing performance. As expected, experience (driver’s learning experience and learning experience related to traffic) was found to be the most important contributory factor in sign cognition. In addition, attention to traffic signs improved the sign-guessing performance of users who provided positive assessments of sign cognition. Although these results seem intuitive, they lead to the conclusion that travelers may be able to improve their own sign guessing capabilities by reviewing samples of traffic signs before travel. Semantic distance (closeness) and confidence in guessing were confirmed as two contributory features for designing better signs. Cultural issues were verified in this study. However, latent psychological factors were not considered in this paper. Based on a newly published paper [45], the need for closure (information needs), risky driving style and anxiety affect road sign comprehension. These issues require further investigation in future work.

We recommend designing symbols that are closely connected to the real environment to avoid these cultural issues. We also recommend using concrete symbols that eliminate semantic distance as a general principle to improve the guessing capabilities of novice road users. This study can provide useful information and recommendations for designing user-friendly traffic signs and effective ways of using them.

## Figures and Tables

**Table 1:** Fifty-four traffic signs selected for the investigation.

Sign Number	Sign Pattern	Sign Number	Sign Pattern	Sign Number	Sign Pattern
**1**	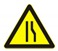	19	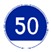	**37 (German)**	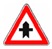
**2**	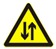	20	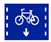	**38 (German)**	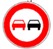
**3**	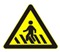	21	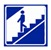	39	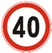
**4**	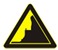	22	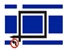	**40 (German)**	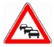
**5**	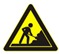	23	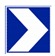	41	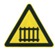
**6**	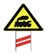	24	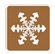	42	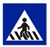
**7**	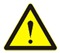	25 (German)	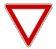	**43 (German)**	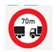
**8**	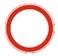	26	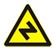	44	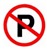
**9**	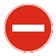	27	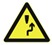	45	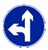
**10**	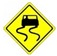	28	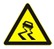	46	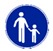
**11**	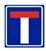	**29 (German)**	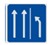	47	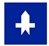
**12**	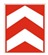	**30 (German)**	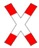	48	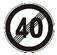
**13**	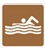	**31 (German)**	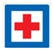	49	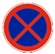
**14 (German)**	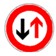	**32 (German)**	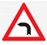	50	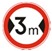
**15 (German)**	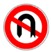	33	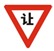	51	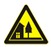
**16**	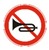	**34**	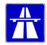	**52 (German)**	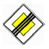
**17**	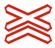	**35 (German)**	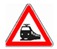	**53 (German)**	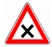
**18**	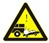	36	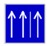	**54 (German)**	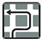

**Table 2:** Rating level for the sign design features.

	Rating	1	2	3	4	5
Feature						
Familiarity		Completely unfamiliar	Relatively unfamiliar	General/ moderate	Relatively familiar	Very familiar
Complexity		Completely simple	Relatively simple	General/ moderate	Relatively complex	Very complex
Confidence in Guessing		Completely unconfident	Relatively unconfident	General/ moderate	Relatively confident	Very confident
Semantic Distance		Completely consistent	Relatively consistent	General/ moderate	Relatively inconsistent	Completely inconsistent

**Table 3:** One-sample Kolmogorov–Smirnov test.

		Guessing Score
N		201
Normal Parameters	Mean	1.52
	Deviation	0.231
Kolmogorov-Smirnov Z		6.104
*p*-value		0.647

**Table 4:** Descriptive statistics of guessability scores for signs in all categories.

	Score	Average Guessing Score	Standard Deviation	Coefficient of Variation	Maximum	Minimum
Types						
Warning (17)		62.88	19.59	31.16	88.33	23.00
Prohibition (15)		57.09	29.49	51.66	99.00	6.00
Mandatory (12)		49.67	27.80	55.97	82.33	6.00
Guide (1)		35.67	NA	NA	NA	NA
Tourist (2)		50.00	12.73	25.46	59.00	41.00
Roadwork (2)		42.50	8.91	86.15	48.80	36.20
Special (5)		9.40	13.01	138.45	31.33	0.33
Total (54)		50.70	28.11	55.44	99.00	0.33

**Table 5:** The rating score of the seven signs that received the lowest guessing scores.

Number	Symbols	Correct Meaning	Guessed Score (%)			The Three Most Frequent Responses
			Mean	Standard Deviation	Coefficient of Variation	
47	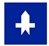	Traffic has priority in the main road	11.67	30.87	264.53	Big rocket? (32%) Go ahead (40%) Main road has the right of passage (6%)
37	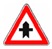	The right of way for the viewer of the sign at the next crossing	11.00	28.17	256.06	Go ahead (52%) Rocket? (30%) Main road has the right of passing (6%)
25	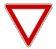	Slow down and yield to pedestrians	6.00	21.10	351.67	Do not know (60%) No entry (24%) Give away (6%)
46	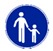	Pedestrians only	6.00	17.23	287.22	Watch out for children (48%) School area (40%) Only for walking (6%)
30	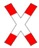	Level crossing	4.00	15.83	395.83	No entry (73%) Accident ahead (11%) Intersection (6%)
52	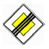	The end of priority road	0.33	2.34	710.00	Do not know (80%) No passing (10%) Turn right (5%)
53	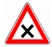	Uncontrolled Intersection ahead, proceed with extreme caution, priority is not assigned.	0.33	2.14	650.00	No entry (60%) Tunnel ahead (15%) Do not know (10%)

**Table 6:** Responses for the eight user factors and the mean guessing performance.

User Factors	Response	Users Number (%)	Guessing Performance (%)	
			Mean	Standard Deviation
Driver’s license training experience	With driver’s license training	79 (39%)	61.06	9.40
	No driver’s license training	122 (62%)	43.98	5.40
Grades	Grade one	68 (34%)	44.57	8.10
	Grade two	73 (36%)	51.45	11.04
	Grade three	60 (30%)	56.71	10.49
Gender	Male	105 (52%)	51.08	11.00
	Female	96(48%)	48.74	11.27
Vehicle ownership	Vehicle-available household	76 (38%)	53.04	10.07
	Vehicle-unavailable household	125 (62%)	49.27	11.05
Attention to the design of traffic signs	Paid attention to traffic signs	98 (48.76%)	54.35	12.03
	No attention to traffic signs	103 (51.24%)	47.23	8.77
Traffic incident experience	Had traffic incident experience	22 (10.94%)	50.98	10.31
	No traffic incident experience	179 (89.06%)	50.66	11.17
Believe that the sign meaning can be guessed only by yourself	Yes	113 (56.22%)	52.04	11.75
	No	88 (43.78%)	48.97	9.87
Living area	Rural areas	94 (46.77%)	48.80	10.74
	Urban areas	107 (53.23%)	51.20	11.00

**Table 7:** ANOVA and Kruskal-Wallis test for the user factors.

Factor	ANOVA Test	
	*F*-Value	Sig
Driver’s license training	266.66	0.000 **
Traffic incident experience	0.16	0.69
	**Kruskal–Wallis test**	
	**χ^2^-Value**	**Sig**
Grade	44.435	0.000 **
Gender	1.59	0.201
Vehicle ownership	8.08	0.008 *
Attention to the design of traffic signs	16.751	0.000 **
Living area	3.276	0.095
Believe that the sign meaning can be guessed only by yourself	2.33	0.55

* significant at the 0.01 level; ** significant at the 0.001 level.

**Table 8:** Pearson correlation analysis among traffic sign features.

Features	Familiarity	Complexity	Confidence in Guessing	Semantic Distance
Familiarity	—			
Complexity	−0.701 **	—		
Confidence in guessing	0.935 **	−0.622 **	—	
Semantic distance	−0.689 **	0.519 **	−0.813 **	—

** Correlation is significant at the 0.001 level (2-tailed).

**Table 9:** Pearson correlation analysis between guessing score and sign features.

Features	Familiarity	Complexity	Confidence in Guessing	Semantic Distance
Familiarity	—			
Complexity	−0.701 **	—		
Confidence in guessing	0.935 **	−0.622 **	—	
Semantic distance	−0.689 **	0.519 **	−0.813 **	—
Guessing Score	0.672 **	−0.423 **	0.820 **	−0.923 **

** Correlation is significant at the 0.001 level (2-tailed).

**Table 10:** The results of improving the design of five signs with low guessing scores.

Original Sign	Improved Sign	Meaning	Rating Score of the Improved Signs		
			Confidence in Guessing (%)	Semantic Distance (%)	Change in Guessing Score (%)
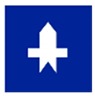	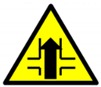	Watch out! Main road has the right of the way	72.2	23.1	28.17→78.4
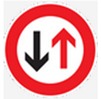	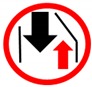	Stop for oncoming vehicles	53.4	32.4	34.67→68.5
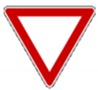	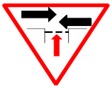	Slow down and yield to others	55.6	36.4	21.10→71.4
